# Effects of Anakinra on Health-Related Quality of Life in a Patient with 1129G>A/928G>A Mutations in MVK Gene and Heterozygosity for the Mutation 2107C>A in CIAS1 Gene

**DOI:** 10.3389/fped.2017.00128

**Published:** 2017-06-07

**Authors:** Gianluigi Laccetta, Maria Tutera, Mario Miccoli, Rita Consolini

**Affiliations:** ^1^Department of Pediatrics, Faculty of Medicine, University of Pisa, Pisa, Italy; ^2^Department of Clinical and Experimental Medicine, Faculty of Medicine, University of Pisa, Pisa, Italy

**Keywords:** mevalonate kinase deficiency, anakinra, health-related quality of life, autoinflammatory diseases, fever

## Abstract

Mevalonate kinase deficiency impairs several aspects of the patient’s quality of life, thus early diagnosis and treatment are required to improve health-related quality of life (HRQOL). A 15-year-old patient with double heterozygosity for the mutations 1129G>A and 928G>A in MVK gene, heterozygosity for the mutation 2107C>A in CIAS1 gene and hyper-IgD syndrome phenotype, has been treated with anakinra with a reduction of 50% in the number of fever episodes per month, a reduction of 33% in the days of fever for each attack and normal blood tests in the intercritical phase. The RAND 36-Item Health Survey has been used for the assessment of HRQOL before and after the treatment with anakinra. The patient’s quality of life showed an overall improvement of 27%; results showed a better improvement in role limitations due to physical health (50%).

## Introduction

Mevalonate kinase deficiency (MKD) is a rare autosomic recessive autoinflammatory disease caused by mutations of the MVK gene on chromosome 12q24, whose clinical spectrum ranges from hyper-IgD syndrome (HIDS) to lethal forms of mevalonic aciduria (MA) (1, 2). HIDS usually manifests before the age of 3 years, and it is characterized by recurrent and lifelong episodes of fever and inflammation lasting 5–7 days, transient arthritis, headache, abdominal pain, vomiting, hepatosplenomegaly, lymphadenopathy, and skin rashes (1). Fever attacks can occur in a cyclical pathway or be provoked by illnesses, injuries, and vaccinations; they are more frequent during childhood and decrease with age (2, 3). According to the International HIDS Database, the percentage of patients with more than 6 febrile attacks per year is about 90% in the first decade of life, 73% in the second decade of life and 50% after the age of 20 years; the percentage of patients with more than 12 febrile attacks per year is 44% in the first decade of life, 24% in the second decade of life, and 18% after the age of 20 years (4). Complete remission with age has never been reported (4). MA is also accompanied by psychomotor retardation, progressive visual impairment, cerebellar ataxia, seizures, myopathies, growth retardation, physical dysmorphisms (frontal bossing, hypertelorism, long eyelashes, triangular-shaped facies), cholestasis, and liver dysfunction (1, 2).

It has been documented that MKD affects 300 people worldwide, with a higher prevalence in the Dutch population (about 1:200,000 affected nationwide); HIDS is the most represented phenotype among affected people (2, 4). More than 120 sequence variants in MVK gene have been reported in patients affected by MKD; genotype–phenotype correlation is only partial, therefore additional genes are suspected to regulate genotype–phenotype correlation (2, 5, 6). In MKD, there is a depletion of 5-phosphomevalonic acid and its downstream products (geranyl pyrophosphate, farnesyl pyrophosphate, and geranylgeranyl pyrophosphate), which are implicated in protein prenylation (2). Cellular isoprenoid deficiency leads to the activation of monocytes, macrophages, RhoA, and Rac1, with subsequent IL-1β hypersecretion and proinflammatory cellular pyroptosis and apoptosis (2). HIDS is usually associated with high serum IgD (72–88% of patients); during flares, C-reactive protein and erythrocyte sedimentation rate are above the normal range, and neutrophil predominant leukocytosis is frequent (2, 4, 7, 8). Urinary mevalonic acid is often elevated, and mevalonate kinase function is reduced (1.8–28% in HIDS patients, less than 0.5% in MA patients); 3% of HIDS patients have also high serum amyloid A (2).

Mevalonate kinase deficiency impairs several aspects of the patient’s quality of life (physical and social functioning, school, career, finance, emotion) with negative consequences on family life, independence, employment status, and daily activities, thus early diagnosis and treatment are required to improve health-related quality of life (HRQOL) (3).

Currently, no therapies have been approved for MKD: colchicine and statins have performed poorly; non-steroidal anti-inflammatory drugs and corticosteroids have also been used with some benefits (1–3). Colchicine provided a complete response in none of the patients from the Eurofever registry and in 2% of patients from a literature review by the Eurofever registry investigators; partial response was achieved by 35% of patients from the Eurofever registry and 18% of patients from the literature review (9). Statins provided a complete response in none of the patients from the Eurofever registry and the literature review by the Eurofever registry investigators; partial response was achieved by 27% of patients from the Eurofever registry and 32% of patients from the literature review (9). Non-steroidal anti-inflammatory drugs provided a complete response in 13% of patients from the Eurofever registry; partial response was achieved by 64% of patients from the Eurofever registry (9). Corticosteroids provided a complete response in 24% of patients from the Eurofever registry and in none of the patients from a literature review by the Eurofever registry investigators; partial response was achieved by 67% of patients from the Eurofever registry and 63% of patients from the literature review (3, 9).

According to Ter Haar et al., 59% of patients treated with etanercept, a TNF-α blocking agent, achieved a partial response, and only one complete response was reported (9). Complete response after etanercept was achieved by 22% of patients from a literature review by the Eurofever registry investigators, and 33% of patients from the same review reported a partial response (3, 9).

Infliximab provided no response among patients from the Eurofever registry (3, 9). Adalimumab provided a complete response in none of the patients from the Eurofever registry and in 33% of patients from a literature review by the Eurofever registry investigators; partial response was achieved by 50% of patients from the Eurofever registry and 33% of patients from the literature review (3, 9).

Tocilizumab, a monoclonal anti-IL-6 selective antibody, has also been promising, according to recent reports (2, 10). Liver transplantation or hematopoietic stem cell transplantation is good therapeutic option for refractory cases of MA (2, 11).

Anti-IL-1-targeting drugs are generating great interest among pediatric rheumatologists for inadequately controlled MKD (1, 3). Anakinra, a short half-life IL-1 receptor antagonist, has been used in both an “on-demand” and a daily prophylactic dosing regimens (2, 12). It has been demonstrated that Anakinra is associated with decreased duration and severity of fever attacks even when given on-demand (≥50% reduction in duration to a maximum of 2 days of fever in 8 of 12 fever episodes), and its effectiveness is higher within 24 h from the beginning of symptoms; conversely, no effects on the frequency of attacks were noted (2, 13). Furthermore, on-demand treatment with anakinra in HIDS patients is a good option to avoid the fever attacks in patients requiring vaccinations (2, 13). According to Rossi-Semerano et al., 30% of patients reported full response with continuous administration of anakinra, and 70% of patients achieved a partial response (2, 12). Anakinra provided complete response in 22% of patients from the Eurofever registry and in 34% of patients from a literature review by the Eurofever registry investigators; partial response was achieved by 67% of patients from the Eurofever registry and 46% of patients from the literature review (3, 9).

It has also been demonstrated that canakinumab, a long half-life monoclonal anti-IL-1β selective antibody, is effective for inadeguately controlled MKD in dosing ranges from 2 to 7 mg/kg every 4–8 weeks (1, 2, 9, 12). According to Rossi-Semerano et al., canakinumab provides full response in about 50% of patients (12). As regards side effects, 90.2% of children treated with anakinra for an autoinflammatory disease presented at least one adverse effect versus 58.8% of children treated with canakinumab; this difference was mainly due to pain at injection site and injection-site reactions (12). Canakinumab provided complete response in 50% of patients from the Eurofever registry and in 67% of patients from a literature review by the Eurofever registry investigators; partial response was achieved by 50% of patients from the Eurofever registry and 33% of patients from the literature review (3, 9).

According to Galeotti et al., partial clinical remission was achieved by seven of nine patients on anakinra and three of six patients on canakinumab; complete clinical remission was achieved by one of nine patients on anakinra and three of six patients on canakinumab (1). The 11 patients who were enrolled in the study by Galeotti et al. were aged 3–30 years; 8 of them suffered from HIDS and 3 of them were MA patients (1). According to Galeotti et al., the number of days with fever decreased from 5 before treatment to 3 after anakinra and to 2 after canakinumab, and no changes were observed in the frequency of fever attacks (1). In the study by Galeotti et al., four patients were switched from anakinra to canakinumab to achieve a more convenient dosing schedule and to avoid injection-site reactions; canakinumab lowered the clinical score more than anakinra in three of the four patients (1).

Rilonacept provided a complete response in none of the patients from the Eurofever registry; one patient from the Eurofever registry reported a partial response after this treatment (3, 9).

We would like to draw attention to the administration of anakinra in a patient with MKD and its effects on HRQOL.

## Case Report

The patient was a 15-year-old Italian boy who was born from healthy, unrelated parents; he came to our attention because of fever associated with abdominal pain, vomiting, and headache. The patient had a history of recurrent fever attacks (38–39°C) since he was 3 months old. Each attack of fever lasted 4–5 days every 2 weeks and was accompanied by chills, fatigue, sore throat, lymphadenopathy, abdominal pain, and vomiting; folliculitis and aphthous stomatitis were often associated. These episodes were usually preceded by a well-recognized phase of malaise with headache and transient arthritis. In the intercritical periods, the patient was totally asymptomatic. He had already been admitted six times because of prolonged fever and diagnosed as tonsillitis for two times, acute otitis media, sinusitis, and pneumonia for two times. The patient was usually bedridden during severe flares; his life was unpleasant: the recurrent fever attacks limited his activities and relationships and interfered with the development of autonomy during childhood because of the increased dependency on caregivers and the decreased participation in peer and school activities. Recurrent severe flares made the high school graduation unachievable for our patient; his mother had very limited career options, and finally she lost her work. Furthermore, a psychological follow-up was even required for the patient and his caregivers.

On clinical examination, the patient presented fever (38.4°C), sore throat, aphthous stomatitis, cervical lymphadenopathy, enlarged tonsils, and hepatomegaly. His height was 170 cm (50th centile), and weight was 53 kg (10th–25th centile). His blood pressure was 109/71 mmHg, heart rate was 108 per minute, and respiratory rate was 20 per minute.

Laboratory findings were unremarkable except for elevated inflammatory indexes (erythrosedimentation rate 115 mm/h, C-reactive protein 182 mg/dL, serum amyloid A 40 mg/L) and high levels of serum immunoglobulins D (564 IU/mL); all bacterial cultures were negative, and urinalysis was normal. No abnormalities were found on chest X-ray, abdominal ultrasound, and echocardiogram. The diagnosis of HIDS was made on the basis of a double heterozygosity for the MVK mutations 1129G>A and 928G>A; the patient also showed heterozygosity for the mutation 2107C>A in CIAS1 gene.

The periodic symptomatology of the patient has never lessened despite the administration of steroids, non-steroidal anti-inflammatory drugs and antibiotics. Thus, we decided to start the treatment with daily subcutaneous injections of anakinra at a dosage of 1 mg/kg. The first injection of anakinra was followed by a clinical and laboratoristic improvement, thus the patient continued the daily administration of anakinra. After a follow-up of 24 months, we have noticed a reduction of 50% in the number of fever episodes per month and a reduction of 33% in the days of fever for each attack. Blood tests became normal in the intercritical phase, differently from the period in which anakinra was not administered.

The RAND 36-Item Health Survey is a 8-item questionnaire that explores physical functioning, bodily pain, role limitations due to physical health problems, role limitations due to personal or emotional problems, emotional well-being, social functioning, energy/fatigue, and general health perceptions; it also presents a single item about the perception of change in health (14). This questionnaire has been used for the assessment of HRQOL in our patient before and after the treatment with anakinra; in our case, the item about the perception of change in health was excluded.

**Figure 1:**
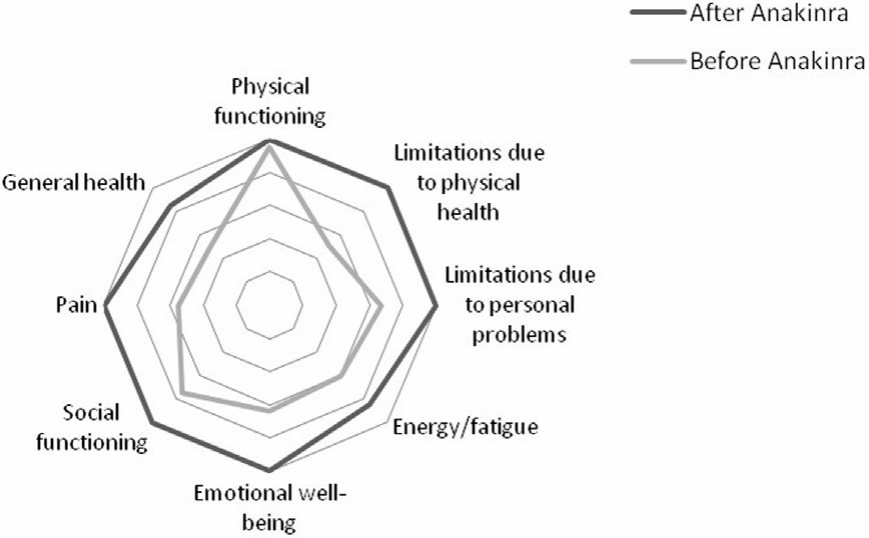
Improvement in each item after the beginning of the treatment with anakinra.

Results showed a better improvement in role limitations due to physical health and in pain (50 and 45%, respectively); improvement in emotional well-being, in general health, and in role limitations due to personal or emotional problems was 36, 35, and 33.3%, respectively. Improvement in energy/fatigue and in social functioning was similar (25% for both the items). Improvement in physical functioning was only 5% as the patient started from 95% and reached 100% in physical functioning after the beginning of the treatment with anakinra. Overall, the patient registered an improvement of 27% in HRQOL after the beginning of the treatment with anakinra. Results are showed in Table 1; Figure 1 shows the improvement in each item after the beginning of the treatment with anakinra.

**Table 1:** Results of the RAND 36-Item Health Survey before and after the treatment with anakinra.

Scale	No. of items	Before anakinra		After anakinra		Improvement (%)
Physical functioning	10	950/1,000	95%	1,000/1,000	100%	+5
Role limitations due to physical health	4	200/400	50%	400/400	100%	+50
Role limitations due to personal or emotional problems	3	200/300	66.7%	300/300	100%	+33.3
Energy/fatigue	4	240/400	60%	340/400	85%	+25
Emotional well-being	5	320/500	64%	500/500	100%	+36
Social functioning	2	150/200	75%	200/200	100%	+25
Pain	2	110/200	55%	200/200	100%	+45
General health	5	250/500	50%	425/500	85%	+35
Total	35	2,420/3,500	69.1%	3,365/3,500	96.1%	+27

## Conclusion

Anakinra has been demonstrated to reduce the number of fever episodes per month and the days of fever for each attack and to improve HRQOL; improvement was more evident in the field of role limitations due to physical health, in pain, and in emotional well-being. In sum, our case confirms the efficacy of anakinra in MKD and points out its efficacy in giving the patient an acceptable quality of life. Positive effects of anakinra on psychological well-being are also pointed out that they are very important for the patient, considering that MKD is chronic disease. Interestingly, our patient was the first to achieve a reduction in the frequency of fever attacks after anakinra.

Furthermore, the patient showed a double heterozygosity for the MVK mutations 1129G>A and 928G>A. 1129G>A (V377I) is the most frequently occurring mutation in MVK gene with an estimated allele frequency of 1:153 (15). The V377I allele has been found exclusively in HIDS patients; however, individuals homozygous for this allele may not be symptomatic (15). Based on the distinct phenotype (symptomatic versus asymptomatic) of two sibs carrying the same homozygous 1129G>A mutation, Messer et al. suggest the existence of modifiers loci controlling the penetrance of HIDS (16). The G-to-A transition at nucleotide 928 (928G>A), resulting in a valine-to-methionine substitution at codon 310 (V310M), is a missense pathogenic variant that has been observed in association with MA (17, 18). The present case report describes a double heterozygous patient for the MVK mutations 1129G>A and 928G>A, which showed an HIDS phenotype.

Our patient also showed heterozygosity for the mutation 2107C>A in CIAS1 gene, which encodes for a protein called cryopyrin; this gene is also known as *NLRP3* (NOD-like receptor 3) (19). Mutations of CIAS1 gene are found in about 70% of patients with a cryopyrin-associated periodic syndromes (CAPS) phenotype (19). Molecular analysis of CIAS1 gene was performed because recurrent fever attacks made us suspect a CAPS phenotype in our patient. CAPS include three autosomal dominant disorders: familial cold autoinflammatory syndrome, Muckle–Wells syndrome, and chronic infantile neurological cutaneous and articular syndrome (CINCA) (19). Cryopyrin is a protein of the inflammasome; mutations in CIAS1 gene are associated with gain of function of cryopyrin and enhanced production of IL-1β (19). The treatment of CAPS is mainly based on subcutaneous anakinra at a starting dosage of 1 mg/kg/day; good results have also been achieved with canakinumab and rilonacept (19–22). According to Aksentijevich et al., among patients with CAPS phenotype, the Q703K (2107C>A) missense change had an estimated allele frequency of 0.04; among Caucasian controls, the 2107C>A nucleotide transversion had an estimated allele frequency of 0.05 (23). Aksentijevich et al. concluded that the allele Q703K is unlikely to be pathogenic because of the similar allele frequency in patients and control cohorts (0.04 versus 0.05, *P* = 0.84) (23). Aksentijevich et al. demonstrated that there is a lack of clear genotype/phenotype correlation for many mutations of CIAS1 gene and mutational severity is not correlated with mutation cluster position, the specific residue mutated or conservation among CIAS1 orthologs, therefore other genetic factors should be involved in determining the disease severity (23). Finally, the presence of the mutation 2107C>A in CIAS1 gene in our patient confirms that the allele Q703K is unlikely to be pathogenic.

## Author Contributions

GL, MT, and RC met the patient to each visit and wrote the case report; MM provided to the construction of the figure and the table.

## Conflict of Interest Statement

The authors declare that the research was conducted in the absence of any commercial or financial relationships that could be construed as a potential conflict of interest.
